# Quercetin inhibits prostate cancer by attenuating cell survival and inhibiting anti-apoptotic pathways

**DOI:** 10.1186/s12957-018-1400-z

**Published:** 2018-06-14

**Authors:** Ashley B. Ward, Hina Mir, Neeraj Kapur, Dominique N. Gales, Patrick P. Carriere, Shailesh Singh

**Affiliations:** Department of Microbiology, Biochemistry and Immunology, Morehouse School of Medicine, Atlanta, GA USA

**Keywords:** Prostate cancer, Quercetin, Apoptosis, Bioflavonoid, Cell survival, Chemoprevention

## Abstract

**Background:**

Despite recent advances in diagnosis and treatment, prostate cancer (PCa) remains the leading cause of cancer-related deaths in men. Current treatments offered in the clinics are often toxic and have severe side effects. Hence, to treat and manage PCa, new agents with fewer side effects or having potential to reduce side effects of conventional therapy are needed. In this study, we show anti-cancer effects of quercetin, an abundant bioflavonoid commonly used to treat prostatitis, and defined quercetin-induced cellular and molecular changes leading to PCa cell death.

**Methods:**

Cell viability was assessed using MTT. Cell death mode, mitochondrial outer membrane potential, and oxidative stress levels were determined by flow cytometry using Annexin V-7 AAD dual staining kit, JC-1 dye, and ROS detection kit, respectively. Antibody microarray and western blot were used to delineate the molecular changes induced by quercetin.

**Results:**

PCa cells treated with various concentrations of quercetin showed time- and dose-dependent decrease in cell viability compared to controls, without affecting normal prostate epithelial cells. Quercetin led to apoptotic and necrotic cell death in PCa cells by affecting the mitochondrial integrity and disturbing the ROS homeostasis depending upon the genetic makeup and oxidative status of the cells. LNCaP and PC-3 cells that have an oxidative cellular environment showed ROS quenching after quercetin treatment while DU-145 showed rise in ROS levels despite having a highly reductive environment. Opposing effects of quercetin were also observed on the pro-survival pathways of PCa cells. PCa cells with mutated p53 (DU-145) and increased ROS showed significant reduction in the activation of pro-survival Akt pathway while Raf/MEK were activated in response to quercetin. PC-3 cells lacking p53 and PTEN with reduced ROS levels showed significant activation of Akt and NF-κB pathway. Although some of these changes are commonly associated with oncogenic response, the cumulative effect of these alterations is PCa cell death.

**Conclusions:**

Our results demonstrated quercetin exerts its anti-cancer effects by modulating ROS, Akt, and NF-κB pathways. Quercetin could be used as a chemopreventive option as well as in combination with chemotherapeutic drugs to improve clinical outcomes of PCa patients.

## Background

Prostate cancer (PCa) affects nearly 70% of men worldwide over the age of 65 and is the second leading cause of cancer-related death following lung cancer in the USA [1]. The prevalence of PCa in the USA is higher than in any other country, suggesting diet and lifestyle play a role in these incidence gaps [2]. Studies showing increased risk of PCa in Asian men moving to the USA and adopting western diet have established convincing association of diet with PCa risk [3, 4]. Solid tumors are surgically removed, but what escapes the surgeon’s knife is of major challenge in cancer treatment and management. Available treatments can increase 5-year survival in early stages of PCa, but the metastatic disease is difficult to manage [5]. Chemotherapy is a classical approach to manage such conditions, but side effects associated with this intervention limit its full utilization. Common chemotherapeutics, however, are toxic and often become ineffective due to development of resistance resulting in disease relapse [6, 7]. Moreover, the efficacy of these drugs is highly compromised due to the indolent nature of PCa cells and oncogenic regulation of molecular processes including apoptosis and cell survival [8–10]. Traditional medicine system uses plant products to treat many disease including cancer, which provide an excellent treatment option with higher benefit-risk ratio [11–13]. Thus, new and efficient anti-cancer agents with potential to enhance efficacy and reduce side effects of conventional therapy are needed for PCa treatment, and plant-based products provide the promising resources for such modality [13].

Quercetin (3, 3′, 4′, 5–7 pentahydroxyflavone) is a bioflavonoid that possesses antioxidant properties and is present in our diet including green vegetables, berries, onions, parsley, legumes, green tea, and citrus fruits [12]. In addition to having antioxidant and gastro-protective effects, quercetin also encompasses anti-inflammatory properties [14–16]. The anti-inflammatory role of quercetin is mainly attributed to its inhibitory effect on inflammatory mediators like nitric oxide, catalase, and pro-inflammatory cytokines TNF-α, IL-6, and IL-1β [15, 17–20]. Quercetin inhibits expression of pro-inflammatory genes by targeting TNF-α-induced recruitment of NF-κB transcription factor to their promoter region [21]. Besides, quercetin also blocks the production of poly-unsaturated fatty acid (PUFA) metabolites associated with inflammatory diseases and cancer progression, by inhibiting PUFA-metabolizing enzyme “lipoxygenase” [22]. Similar anti-inflammatory response of quercetin was observed in treating chronic prostatitis [23–27]. It inhibits carcinogenicity either alone or in combination with chemotherapeutic agents [25, 28]. The anti-cancer effects of quercetin have been shown in several cancers such as breast, cervical, pancreatic cancers, and prostate [12, 25, 29, 30]. In addition to this, 36% decrease in PCa risk for men in the highest quartile of quercetin consumption was reported in a case control study compared to those in the lowest quartile of intake [31]. However, molecular mechanism of quercetin action on cancer prevention and treatment is not fully defined. In this study, using human PCa cell lines, we have defined the change in molecular profile and hence the anti-cancer effect, induced by quercetin in PCa.

## Methods

### Cell culture and reagents

Human PCa cell lines LNCaP, DU-145, and PC-3 were obtained from American Type Culture Collection (ATCC). LNCaP, DU-145, and PC-3 cells were cultured in Roswell Park Memorial Institute (RPMI), 1640 medium at 37 °C with 5% CO_2_ and supplemented with 10% fetal bovine serum (FBS; Hyclone, Logan, UT, USA). Normal prostate epithelial cells (PrEC), with materials purchased from ATCC, were cultured in basal medium with cell growth kit containing the following: 6 mM L-glutamine, 0.4% Extract P, 1.0 μM epinephrine, 0.5 ng/mL rhTGFα, 100 ng/mL hydrocortisone, 5 μg/mL rh insulin, and 5 μg/mL Apo-transferrin. All the cell lines were checked and confirmed as mycoplasma-free. Quercetin dihydrate (Sigma Aldrich, St. Louis, MO, USA) was dissolved in 100% dimethyl sulfoxide (DMSO; Corning, Manassas, VA, USA) before further dilutions. Working concentrations did not exceed DMSO of 0.2%.

### Determination of cell viability

Cells were seeded at a density of 1 × 10^4^ cells per 100 μL in a 96-well plate. After a 24-h incubation growth period at 37 °C, cells were treated with various concentrations of quercetin (5, 10, 20, 40, 80, and 160 μM) at time periods of 24, 48, and 72 h in 2% RPMI. Next, 20 μL of 5 mg/mL thiazolyl blue tetrazolium bromide (MTT; Acros Organics, Fair Lawn, NJ, USA) dissolved in Dulbecco’s phosphate buffered saline (DPBS; Corning, Manassas, VA, USA) was added and plates were incubated at 37 °C for 3 h. A volume of 200 μL of DMSO was added to dissolve formazan crystals formed by viable cells after removing media. Optical density (O.D.) was measured at 570 nm in a spectrometer reader (BMG FLUOstar OPTIMA microplate reader, Cary, NC, USA). Percent cell viability was determined with respect to control. All concentrations were tested in triplicates, and the experiment was repeated three times.

### Apoptosis, reactive oxygen species, and mitochondrial membrane potential analysis by flow cytometry

PCa cells were cultured in a 6-well plate with 5 × 10^5^ cells/well and incubated for 24 h. Cells treated with quercetin were harvested at specified time points, washed in fluorescence-activated cell-sorting (FACS) buffer prepared with 2% FBS in PBS, followed by manufacturer’s instructions for FITC-Annexin V Apoptosis Detection Kit with 7-AAD (BioLegend, San Diego, CA, USA). Data was acquired using flow cytometry (EMD Millipore Guava easyCyte flow cytometer, USA).

For mitochondrial membrane potential and reactive oxygen species (ROS), PCa (5 × 10^5^) cells were seeded in 6-well plates for 24 h before quercetin treatment (40 μM). MitoProbe JC-1 Assay Kit (M34152) was used as an indicator of mitochondrial outer membrane potential following manufacturer’s recommendations (Molecular Probes, Life Technologies, Eugene, OR, USA). After quercetin treatment, PCa cells were scraped and transferred to 1 mL of PBS. Positive and negative substrates for membrane potential were added and incubated at 37 °C for 20 min. Cells were washed twice with 1× PBS and finally resuspended in 500 μL of PBS. JC-1 dye exhibits potential dependent accumulation in the mitochondria by fluorescence shift from green to red. A shift from red-green aggregates was measured with excitation at 488 nm to observe in comparison to controls.

For oxidative stress estimation, ROS-ID Total ROS detection kit for microscopy and flow cytometry was used by following manufacturer’s protocol (Enzo, Farmingdale, NY, USA). PCa cells were washed with 2% FACS buffer and centrifuged for 5 min at 400×*g* at room temperature. The cells were finally resuspended in 500 μL of ROS detection reagent and stained for 30 min at 37 °C in the dark before acquiring data using Guava easyCyte flow cytometer.

### Antibody microarray analysis

Protein lysates were collected by using Cancer Signaling Phospho Antibody Microarray (PCS248) with four slides containing 269 antibodies to be scanned and signal quantified by Axon GenePix 4000B microarray scanner (Molecular Devices, Sunnyvale, CA, USA). Average signal intensity of the replicate spots was normalized to the median signal of the slide for each antibody. Fold changes in P/N ratio (phosphorylated/total protein) were calculated by dividing normalized average signal intensities for quercetin-treated samples by untreated controls. CIMminer platform (https://discover.nci.nih.gov/cimminer/home.do), developed by the Genomics and Bioinformatics Group at the National Cancer Institute, was used to generate a heat map based on the data obtained.

### Western blot analysis

Protein isolated (50 μg) from PCa cells quantified by the Pierce BCA Protein Assay Kit (Thermo Scientific, USA) was resolved on sodium dodecyl sulfate (SDS)-polyacrylamide gel electrophoresis and transferred to polvinylidene fluoride membrane (PVDF; Bio-Rad, Hercules, CA, USA) using a semi-dry transfer system (Bio-Rad, Hercules, CA, USA). PVDF membranes with proteins were blocked for approximately 1 h at room temperature in 5% non-fat milk made in 1× PBS Tween 20 (Fisher Scientific, Faith Lawn, NJ, USA). The membranes were incubated with primary antibodies (1:1000 dilution in 5% non-fat milk PBST) at 4 °C overnight followed by the horseradish peroxidase (HRP)-conjugated secondary antibody anti-mouse IgG (RD, HAF018) and anti-rabbit IgG (RD, HAF058) at room temperature. Rabbit monoclonal BIM (C34C5), BAX (D2E11), PARP (46D11), and PUMA (D30C10) were purchased from Cell Signaling. Rabbit polyclonal anti-*p*GSK-3β Ser9 (D3A4), anti- *p*NF-kB Ser536 (ab #3031), and monoclonal mouse GAPDH (D4C6R) were purchased from Cell Signaling. Protein bands were developed using Trident femto western HRP substrate series (GeneTex, Irvine, CA, USA), and images were captured using the ImageQuant LAS 4000 (GE Healthcare Life Sciences, UT, USA). The blots were re-probed each time to stain with a different primary antibody after stripping with Restore PLUS western blot stripping buffer (Thermo Scientific) for 8 min at room temperature. GAPDH was used as a loading control to ensure equal loading. Image J software (https://imagej.nih.gov/ij/) was used to semi-quantify the optical density and normalized to internal control GAPDH.

### Statistical analysis

The mean and standard error (SEM) were calculated for each experimental and control group. Expression of proteins as well as flow cytometry results in PCa cell lines were compared using a two-tailed Student *t* test between the groups and a two-way ANOVA for cell viability analysis. A P/N ratio was performed for normalizing antibody microarray results. Significant differences between the groups were calculated at alpha level of 0.05, and results are shown as mean ± SEM of three independent experiments.

## Results

### Quercetin decreases cell viability and induces apoptosis in PCa cells

**Fig. 1:**
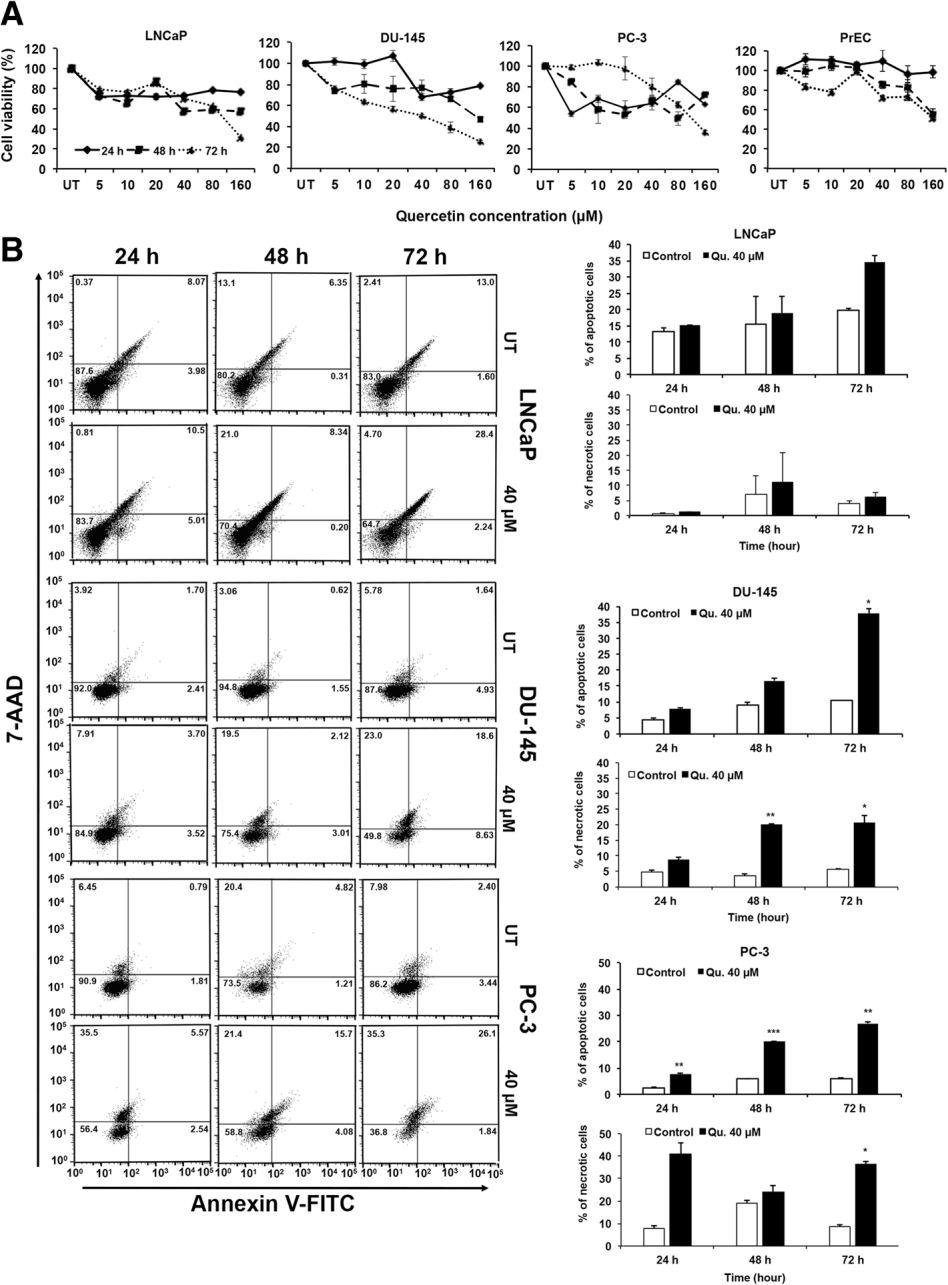
Quercetin reduces cell viability and induces apoptosis in PCa cells. Normal prostate epithelial cells PrEC and PCa cells (LNCaP, DU-145, PC-3) were treated with quercetin, and MTT assay was used to determine cell viability (**a**). The EC_50_ was calculated from the equation of the line of best fit. PCa cells were treated with 40 μM (EC_50_) of quercetin, and vehicle-treated controls were stained with FITC-conjugated Annexin V and 7-AAD. Data was acquired by FACS and analyzed using FlowJo software (**b**). Dot plot shows percent (%) of early apoptotic (lower right quadrate) and late apoptotic cells (right upper quadrate). Apoptosis induced by quercetin (shown in top bar diagram) as well as induced necrotic cells (bottom bar diagram) were measured by paired *t* test to show significance compared to controls

Quercetin treatment significantly decreased cell viability of PCa cell (LNCaP, DU-145, and PC-3) in a time- and dose-dependent manner, without affecting normal prostatic epithelial cells (PrEC) (Fig. 1a). We subsequently determined if the decrease in cell viability was associated with induction of apoptosis. Results from our apoptosis assay showed 40 μM of quercetin treatment for 24, 48, and 72 h increased the percentage of Annexin V-stained FITC-positive cells representing early apoptotic cells by nearly double compared to controls (Fig. 1b). Maximum apoptosis (early and late phase) was observed in LNCaP (30.64%), followed by PC-3 (27.9%) cells and DU-145 (27.2%) after a 72-h treatment with quercetin (40 μM). Similarly, necrotic cells were observed after 72 h with quercetin treatment for LNCaP (4.7%), DU-145 (23%), and PC-3 (35.3%). Our results clearly suggest induction of apoptosis by quercetin in PCa cells followed by secondary necrosis over a period of time. Further experiments were done using a dose of 40 μM quercetin.

### Quercetin modulates ROS production and mitochondrial membrane potential (∆Ψ_m_) in PCa cells

**Fig. 2:**
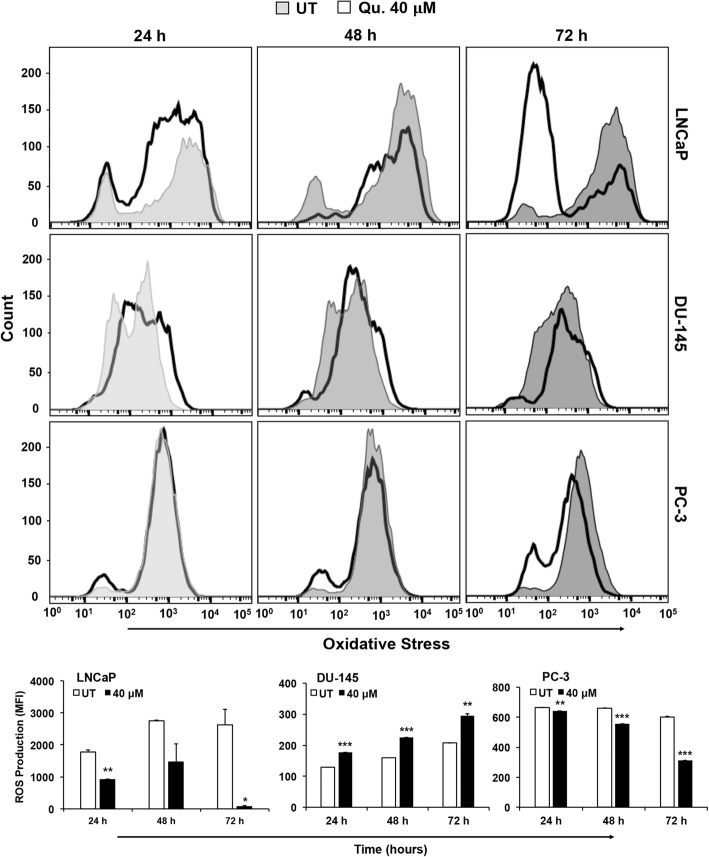
Quercetin impacts reactive oxygen species production in PCa cells. PCa cells were treated with 40 μM of quercetin and were incubated with ROS detection mix. Data was acquired by FACS and analyzed by FlowJo software. Histogram shows the number of ROS producing cell (*y*-axis) and intensity of ROS (*x*-axis). Open histogram represents ROS number and intensity of ROS production in response to quercetin and gray histogram represents vehicle-treated control. Oxidative stress levels were quantified by paired *t* test (depicted in bar diagram) between treated and vehicle-treated control

**Fig. 3:**
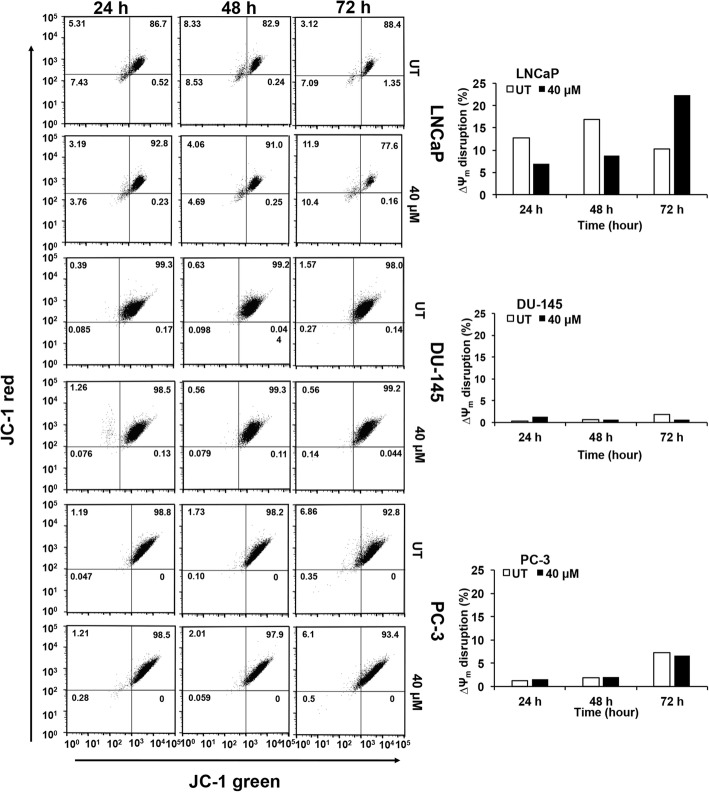
Quercetin modulates mitochondrial membrane potential (∆Ψ_m_) in PCa cells. PCa cells were treated with quercetin and were stained with JC-1 dye, to measure the mitochondrial membrane potential by flow cytometry. FlowJo software was used to analyze change in mitochondrial membrane potential (∆Ψ_m_). JC-1 green aggregates, depicted in lower left quadrant, represent a depolarization shift of the ∆Ψ_m_, and cell death from JC-1 red aggregates show healthy cells (top right quadrant). The bar diagram quantifies mitochondrial membrane potential aggregates between treated and vehicle-treated control

Flow cytometric analysis showed significant increase in level of oxidative stress in DU-145 cells at all time points (24, 48, and 72 h) in response to quercetin. However, LNCaP and PC-3 cells showed decrease in ROS production in comparison to untreated cells, with maximum reduction at a 72-h treatment. Basal level of oxidative stress was higher in LNCaP and PC-3 cells relative to DU-145 cells (Fig. 2). Since mitochondrial membrane integrity is sensitive to cellular ROS, we assessed disruption of mitochondrial membrane potential (MtMP) in PCa cells after quercetin treatment. LNCaP cells exposed to 40 μM quercetin at 72 h had a decrease in MtMP suggesting mitochondrial disruption as indicated by decrease in red/green fluorescence intensity ratio (Fig. 3). Statistical significance was not seen in the more aggressive cell lines, DU-145 and PC-3.

### Quercetin targets apoptotic mechanisms to induce PCa cell death

**Fig. 4:**
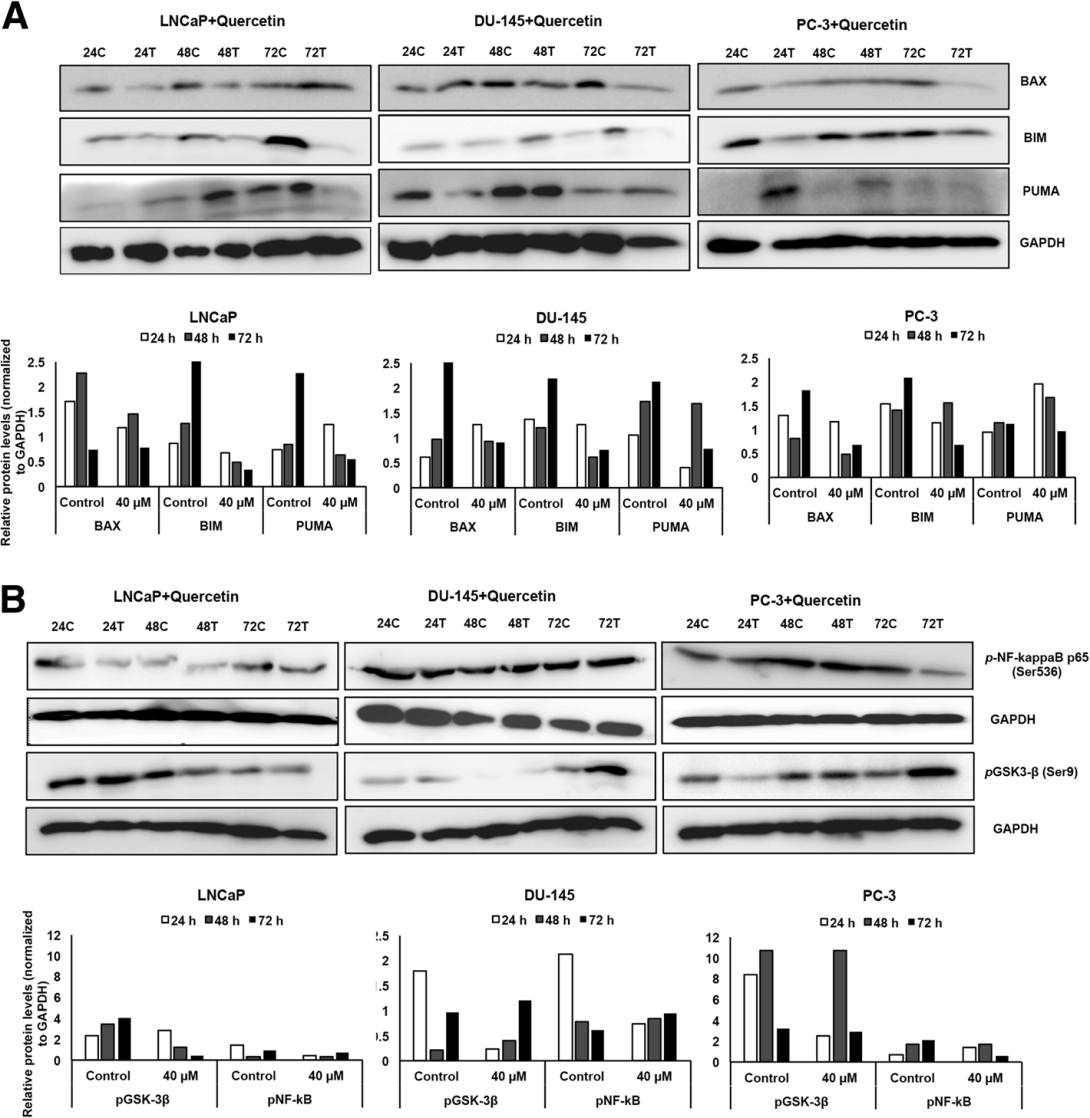
Quercetin modulates apoptotic and survival proteins in PCa cells. Total protein lysate from quercetin-treated PCa cells were resolved on SDS-PAGE and detected using antibodies against apoptotic (**a**) and survival molecules (**b**). Immuno-intensity was quantified using ImageJ software and normalized with GAPDH

Evasion from apoptosis has been identified as a hallmark of cancer cells [24]; however, quercetin inhibits this evasion by upregulating apoptotic machinery in PCa cells. Androgen-sensitive LNCaP cells with wild-type p53 showed decreased BAX and BIM expression at all three time points while PUMA expression was increased at 24 h followed by a decrease at later time points (Fig. 4a). In androgen-independent (DU-145) cells with mutated p53, quercetin treatment increased BAX levels at 24 h with subsequent decrease at 48 and 72 h, whereas BIM decreased significantly after 48 h. However, PUMA significantly decreased at 24 h (Fig. 4a). PC-3 cells, which represent androgen-independent PCa cells lacking p53, showed significant decrease in BAX and BIM expression after 24 h, but increased expression was observed in PUMA at 24 and 48 h (Fig. 4a).

**Fig. 5:**
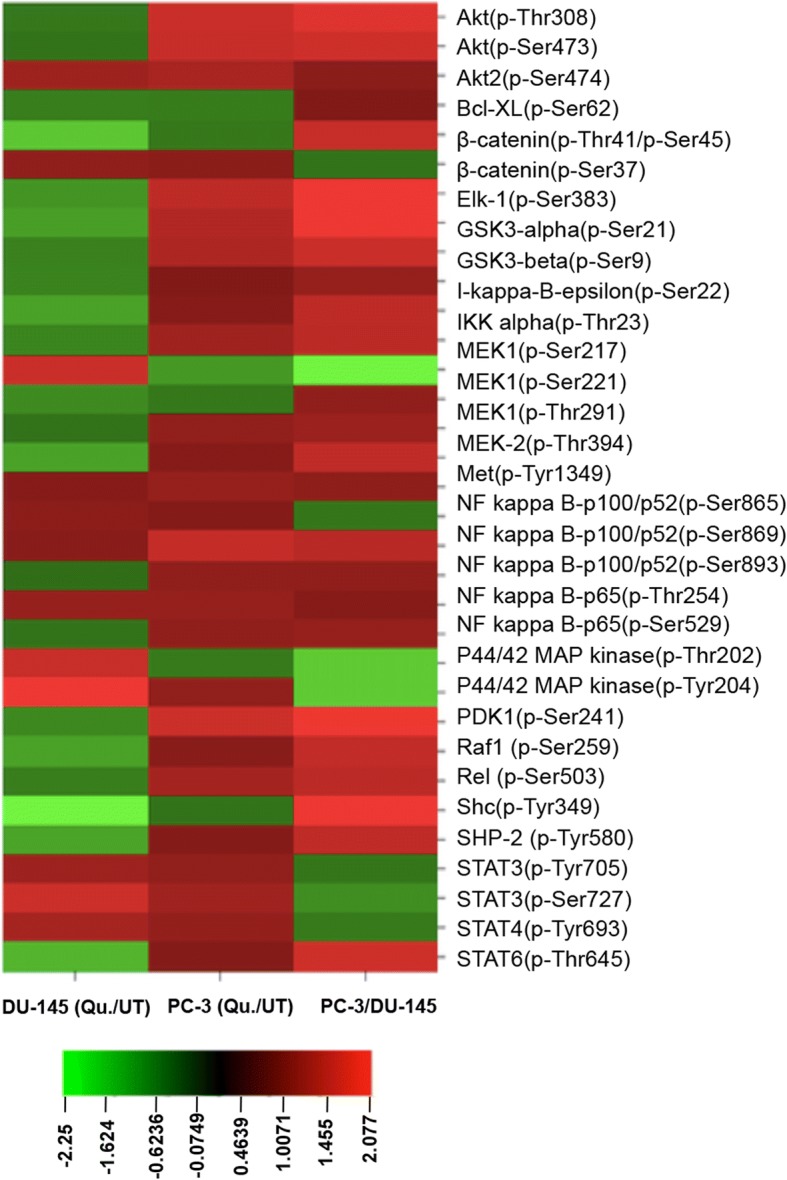
Quercetin regulates signaling molecules involved in PCa cell survival and apoptosis. Antibody microarray was performed on quercetin (Qu.)-treated and untreated (UT) DU-145 and PC-3 cell lines. The heat map was generated from normalized intensity data using CIMminer tool (Genomics and Bioinformatics group, NIH). The heat map represents fold change in phosphorylation status of cell survival and pro-/anti-apoptotic molecules in quercetin-treated DU-145 and PC-3 cells. Each cell in the heat map shows ratio of phosphorylated (P) to non-phosphorylated (N) protein in treated vs untreated sample. Red indicates increase while green represents decrease in phosphorylation of signaling molecules, and intensity of color depends on degree of phosphorylation

To define the underlying molecular mechanism targeted by quercetin, we screened over 250 different proteins in cancer cell signaling using antibody microarray. Changes in phospho-proteomic profile of molecules involved in cell survival and apoptosis were observed in response to quercetin treatment compared to controls (Fig. 5). DU-145 and PC-3 cells showed a notable change in phosphorylation status of key molecules of MAPK and Akt pathways after 48 h quercetin treatment. Androgen-independent DU-145 cells with mutated p53 showed more than two-fold decrease in phosphorylation of β-catenin (p-Ser37) and Shc (p-Tyr349), implicating downregulation of pro-tumorigenic PI3K/Akt pathway. However, components of cancer signaling MAPK/Erk pathway were significantly increased as observed by increased phosphorylation status of MEK1(p-Ser221) and p44/42 MAPK (p-Thr202 and p-Tyr204) (Fig. 5). Contrasting results were obtained in androgen-independent PC-3 cells treated with 40 μM quercetin, which showed significant increase in activation of PI3K/Akt pathway. Key molecules of PI3K/Akt pathway, which are significantly phosphorylated in PC-3 cells lacking p53 include PDK1(p-Ser241), Akt(p-Thr308 and Ser473), PTEN (p-Ser380/Thr382/Thr383), GSK-3β(p-Ser9), NF kappa B-p105/p50(p-Ser893), and BAD(p-Ser112) (Fig. 5). Antibody microarray results also showed increase in phosphorylation of transcription factor Elk-1 at serine 383 (p-Ser383), which is one of the main downstream regulators of MAPK/Erk pathway, in PC-3 cells. Further, differential activation of pro-apoptotic and cell survival pathways in different PCa cells were examined by phospho-activation of key upstream molecules, GSK-3β (p-Ser9) and NF-κB-p65 (p-Ser 536) using western blot analysis (Fig. 4b). Both androgen-sensitive (LNCaP) and androgen-independent (DU-145 and PC-3) PCa cells showed significant decrease in pNF-κB (p-Ser 536), though after different intervals of quercetin treatment. LNCaP and DU-145 cells showed significant decrease in phosphorylation of pNF-kappa B after 24 h, while PC-3 showed decrease after 72 h (Fig. 4b). Another key signaling molecule, GSK-3β, showed marginal increase in phosphorylation at Ser 9 initially after 24 h but decreased significantly thereafter in subsequent days (48 and 72 h) (Fig. 4b). However, both androgen-independent cells (DU-145 and PC-3) showed significantly decreased phosphorylation of GSK-3β (Ser 9) at 24 h with subsequent increase after 72 h (Fig. 4b).

## Discussion

Chemotherapy is often used to treat advance PCa, either alone or in combination with therapeutic modalities. However, patients often develop resistance that can lead to poor therapeutic response and disease relapse. Therefore, new agents are urgently needed to improve therapeutic outcome of PCa with minimal side effects. In this regard, natural agents such as flavonoids have been on the rise to determine their anti-cancer properties in different cancer types. The major focus of this study was to define quercetin-induced alterations in molecules that cancer cells often use to evade apoptosis.

Quercetin has been reported to have a therapeutic effect against PCa [12, 25, 30]. It can modulate the variety of processes involved in cancer progression and metastasis. Quercetin is used for treatment for prostatitis, which can be an indicator for PCa development [23, 32, 33]. Several studies have recognized the anti-inflammatory and anti-proliferative effects of quercetin on various human cancer cell lines [34–36]. Apoptosis maintains tissue homeostasis; however, cancer cells develop mechanisms to elude cell death [37, 38]. PCa cells exposed to quercetin showed more accumulation of dead cells with increasing time and dose while primary prostate epithelial cells were not as sensitive. The data favors the conditions that quercetin treatment regime will be associated with fewer side effects. We show the molecular mechanisms of quercetin-induced cell death in early and advanced PCa cells.

Androgens have been known to play a key role in PCa progression [39, 40]. Androgen deprivation therapy (ADT) is the first treatment given to the patients with androgen-dependent disease [41]. Unfortunately, relapse generally occurs within 12–18 months, leaving patients with castration-resistant prostate cancer (CRPC) [42]. Few studies have implemented natural polyphenolic compounds as an alternative to counteract the function of AR, either indirectly or directly by targeting androgen-regulated genes [39, 43]. Quercetin modulates the components of insulin-like growth factor signaling and induces intrinsic as well extrinsic pathway-mediated apoptosis in androgen-independent conditions [44]. Further, quercetin also induces c-jun/sp1-mediated downregulation of AR expression and activity in PCa cells [45]. Furthermore, quercetin attenuated the transcriptional output of AR by repressing its expression in androgen-responsive PCa cells [39]. These findings commonly focused on using PCa cell lines with wild-type AR expression (LNCaP) and need further observations in cell lines that has mutated (DU-145) and lac AR (PC-3) to understand mechanistic view. Further, quercetin also antagonizes an aberrant AR signaling by targeting the splice factor hnRNPA1 that promotes AR-V7 expression, which is one of the reasons behind the CRPC development [46]. Thus, quercetin resensitizes the resistant PCa to anti-androgen therapy. This inhibitory effect of quercetin on AR signaling implicates its promising role as a chemopreventive agent or as an adjunct to existing therapy for PCa.

Under normal physiological conditions, excessive reactive oxygen species (ROS) are detrimental. Cancer cells have higher ROS levels; however, they optimize multiple signaling mechanisms and learn to use this to support carcinogenesis [47–51]. Quercetin treatment disrupts this new achieved ROS balance in PCa cells either by acting as an antioxidant or as a pro-oxidant depending upon the oxidation status of the cells. In PCa cells that have high basal level of ROS and lack PTEN (LNCaP and PC-3), quercetin serves as an anti-oxidant, whereas in DU-145 cells that have more reductive environment, it serves as a pro-oxidant. Interestingly, it is cytotoxic to all three PCa cell lines irrespective of its effects on ROS generation or the mode of induced cell death. Quercetin treatment can increase ROS levels due to peroxidase-catalyzed oxidation or by lowering intracellular pool of glutathione (GSH) [52]. Quercetin can react with ROS forming harmful quinones [53] that are scavenged by GSH and ultimately leading to depletion depending on the GSH levels of cells [54, 55]. Quercetin-generated free radicals could lead to oxidative damage of nucleic acids, lipid peroxidation, and cell death as reported in human hepatocytes and epithelial cell lines [54, 55]. It could also induce apoptosis via AMPK-α or COX-2 signaling pathway [56]. ROS levels could be associated with apoptosis, p53, or RAS activation; NAD(P)H oxidase system; and mitochondrial integrity. Opposing effects of quercetin on ROS levels consequently reflect in its differential effect on the on MAPK, Akt, and NF-κB pathways in the two androgen-independent PCa cell types that inherently have low levels of activated Raf, MEK, and ERK.

Increased ROS levels, as observed after quercetin treatment of DU-145 cells, could induce Raf/MEK/ERK activation in ligand-dependent as well as in ligand-independent manner in these cell types. This is supported by the microarray data where DU-145 cells have increased MEK1 activation while PC-3 cells do not mirror this effect. Also, Raf1 and MEK2 molecules are inactivated. The upstream molecules of the pathways MEK1 (p-Ser221) and p44/42 MAPK (p-Thr202 and p-Tyr204) show increased phosphorylation and hence activation of the MEK1. However, Elk-1(p-Ser383), which is the downstream target of this pathway, showed reduced phosphorylation. This suggests either the MAPK signaling cascade is blocked at a downstream step or quercetin also activates certain phosphatase activity. Although increased Raf/MEK/ERK pathway is associated with proliferation and drug resistance in advanced PCa cells (PC-3 and DU-145), increase in this pathway after introduction of functional p53 is associated with increased response to chemotherapeutic drugs. Quercetin treatment results in reduced levels of activated Akt pathway molecules that significantly contributes to the reduced survival of these cells. Also, suppressed PI3K/Akt removes the inhibitory effect from Raf/MEK/ERK pathway further supporting the above observation. Whereas, in PC-3 cells, PDK-1/Akt pathway is active, which imposes negative regulation on the MEK pathway.

Treatment with quercetin also increases phosphorylation of Ser-9 residue of GSK-3β (LNCaP, 24 h; DU-145, 72 h; PC-3, 72 h), a critical downstream effector of PI3K/Akt pathway. This phosphorylation at Ser9 inactivates GSK-3β, which in turn limits BAX activity. This also phosphorylates, stabilizes, and hence promotes nuclear translocation of β-catenin, which in turn transcribes tumorigenic genes, thereby regulating myriad of tumorigenic effects through Wnt/β-catenin and other associated pathways [57–59]. However, contrary to the expectation based on GSK-3β phosphorylation status, β-catenin phosphorylation at Ser37 was significantly reduced after quercetin treatment. This implies that quercetin blocks the pro-tumorigenic Wnt/β-catenin signaling. On the other hand, this also reduces GSK-3α and GSK3β phosphorylation (more active) and therefore increased phosphorylation of β-catenin. However, β-catenin Ser37 phosphorylation is reduced suggesting a phosphatase activity.

Both Raf/MEK/ERK and PI3K/Akt pathways interact with p53 and thereby control activity and localization of BIM, BAK, BAX, PUMA, and NOXA. Also, irrespective of the source, ROS could disrupt mitochondrial membrane proteins and hence the organelle integrity [60, 61]. Androgen-sensitive PCa cells with wild-type p53, LNCaP, showed a decrease in BAX and BIM at all three time points while PUMA increased at 24 h, followed by a decrease at later time points with disruption of MtMP in LNCaP cells at 72 h. In androgen-independent PCa cells with mutated p53 (DU-145), quercetin treatment increases cellular BAX levels whereas PUMA and BIM increased, respectively at 24 and 48 h followed by a decrease at following time points. PC-3 cells which represent androgen-independent PCa cells lacking p53 showed increase in PUMA (24 and 48 h), whereas BAX and BIM decreased after 24 h. Phosphorylation of BCL-xL at Ser62 is reduced in both DU-145 and PC-3 that negatively regulates its anti-apoptotic function. This pro- and anti-apoptotic Bcl-2 family of proteins governs the mitochondrial integrity (mitochondrial outer membrane potential, MtMP). BIM can trigger mitochondrial depolarization by stimulation of BAX and BAK oligomerization whereas PUMA can affect the depolarization by inhibiting anti-apoptotic Bcl-2 family members. However, only LNCaP, but not DU-145 and PC-3 cells, showed disruption of MtMP. This suggests that quercetin induces apoptosis by intrinsic pathway in early stage PCa cells whereas mitochondrial perturbation is minimal in advanced PCa cells. Nonetheless, significant accumulation of necrotic cells at 48 and 72 h in DU-145 and PC-3 is observed, suggesting an acute response to quercetin. Therefore, necrosis could be the major cell death mechanism induced by quercetin in these advanced PCa cell types.

Quercetin treatment, however, led to an increased phosphorylating activity of Akt as seen by increased phosphorylation of IKKα (Thr23) as well as IκB-ε phosphorylation (Ser22), which promotes NF-κB activation. Increased phosphorylation at Thr254 is associated with reduced binding of IKKB and hence activation of NF-κB activity. Phosphorylation at Ser529, known to be targeted by IL-1β or TNFα-activated casein kinase 2, implies increased transactivation potential in a gene-specific manner. Phosphorylation on Ser536 could be mediated by various kinases involved in transactivation of NF-κB-targeted genes by acetylation at K310 [62]. While IKKα-mediated phosphorylation at Ser865 and Ser869 as well as increased phosphorylation at Ser893 (cyclin-dependent kinase) promotes processing of p100 [62], the stability of p105 subunit (decreased NF-κB activation) is increased with Ser907 phosphorylation [63] after quercetin treatment. Phosphorylation at this site is mediated by GSK-3β but could represent the pre-phosphorylated molecules as GSK-3β is inactivated in these cells after quercetin treatment. Interestingly, p105 negatively regulates MAPK pathway, which is evident in our results. Quercetin treatment inhibits MEK1 activity by phosphorylation.

Quercetin treatment affects NF-κB activation in PCa cells albeit differentially. There was a remarkable decrease in the phosphorylation of NF-κB at serine 536 albeit at different time points in PCa cell lines. This IKK-mediated phosphorylation activates the canonical NF-κB pathway and is also required for nuclear translocation and acetylation of RelA/p65 and hence activation of NF-κB. Overall reduced activity of NF-κB would affect regulation of anti-apoptotic proteins including Bcl-2 and Bcl-xL [64–68]. Cells with mutated p53 (DU-145) showed reduced NF-κB activation; however, it is apparent from our data that quercetin marginally (based on densitometrin analysis) activates NF-κB after a 24-h treatment, only in p53-null (PC-3) cells as would be expected due to mutual inhibitory effects of p53 and NF-κB [69]. Activated NF-κB in cancer cells is more commonly associated with tumorigenesis and mainly exerts its oncogenic potential by inhibiting apoptosis [70, 71], stimulating cell proliferation [72], and promoting migration and invasion phenotype [73]. However, the end result of activated NF-κB is very much dependent on the coactivators and corepressors including the DNA-binding proteins and transcription factors active in the cells. More and more anti-cancer effects of NF-κB activation are also being reported. Studies involving knock down of IKK-α, IKK-β, and IKK-γ have shown the anti-angiogenic significance of NF-κB activation [74–79]. It is clear from these studies that IKK complex is involved in activating canonical NF-κB pathway, which may have more diverse roles. Besides this interesting aspect of initial NF-κB activation after 24 h of quercetin treatment, overall effect of prolonged exposure of PC-3 cells with quercetin resulted in net reduction in NF-κB activity. Thus, quercetin may be exploiting the pro-apoptotic function of NF-κB pathway. Alternatively, the activated NF-κB could be a response of cancer cells to resist quercetin-induced cell death. If this were true, therapeutic strategies targeting NF-κB pathway in combination with quercetin would dramatically improve patient survival.

## Conclusion

The goal for the study was to determine any existing anti-cancer properties of quercetin that merited results beneficial to a clinical setting. In conclusion, these results show the effect of quercetin on cancer cell signaling and could potentially serve as a mechanistic view of cell death. Our findings suggest quercetin induces cell death in malignant cells, without affecting normal prostate cells, and simultaneously decreases cell survival in PCa cells of different genetic makeup. Moreover, the effect of quercetin on cell viability and programmed cell death was highest in highly aggressive and AR-negative PC-3 cell followed by cells with mutated AR (DU-145) and LNCaP, which is AR-positive and less aggressive suggesting that quercetin is effective in AR responsive as well as CRPC condition. Quercetin capabilities to target PCa cell with varied AR status were accomplished by modulating ROS production and interfering with MAPK, Akt, and NF-κB signaling pathways. In addition to these, many influencing factors that exist in the tumor microenvironment such as ROS and survival molecules play an intricate role in PCa development, progression, and ability to metastasize. Thus, our work highlights the potential of quercetin as a chemo-preventive agent as well as a neo-adjuvant or adjuvant to improve efficacy of conventional therapeutics This should be followed by further investigation in an in vivo model to determine doses of quercetin required and favorable for optimal chemoprevention and therapeutic effects.
